# Cognitive vergence and pupillary responses as functional oculomotor signatures to differentiate AT(N) biological profiles in older adults with mild cognitive impairment

**DOI:** 10.1007/s11357-026-02487-2

**Published:** 2026-08-28

**Authors:** Ricardo Martínez-Flores, Isabel Martín-Sobrino, Neus Falgàs, Oriol Grau-Rivera, Marc Suárez-Calvet, Carlos Cristi-Montero, Agustín Ibañez, Aida Fernández-Lebrero, José Contador, Irene Navalpotro-Gómez, Albert Puig-Pijoan, Hans Supèr

**Affiliations:** 1 Institute of Neurosciences, University of Barcelona (UBNeuro), Barcelona, 08035 Spain; 2 Vision and Control of Action Group, Department of Cognition, Development and Educational Psychology, University of Barcelona, Barcelona, 08035 Spain; 3 Alzheimer’s Disease and Other Cognitive Disorders Unit, Neurology Service, Hospital Clínic de Barcelona, Fundació de Recerca Clínic Barcelona-IDIBAPS, Barcelona, Catalonia Spain; 4 Universitat de Barcelona, Barcelona, Spain; 5 Centro de Investigación Biomédica en Red en Enfermedades Neurodegenerativas (CIBERNED), Madrid, Spain; 6 Barcelonaβeta Brain Research Center (BBRC), Pasqual Maragall Foundation, Barcelona, Spain; 7 Hospital del Mar Research Institute, Barcelona, Spain; 8 Centro de Investigación Biomédica en Red de Fragilidad y Envejecimiento Saludable (CIBERFES), Instituto de Salud Carlos III, Madrid, Spain; 9 Servei de Neurologia, Hospital del Mar, Barcelona, Spain; 10 IRyS Group, Physical Education School, Pontificia Universidad Católica de Valparaíso, Valparaíso, Chile; 11 Center for Interdisciplinary Research in BiomedicineBiotechnology and Well-Being (CID3B), Universidad Católica de Valparaíso, Valparaíso, Chile; 12 Latin American Brain Health Institute (BrainLat), Universidad Adolfo Ibáñez, Santiago, Chile; 13 Global Brain Health Institute, Trinity College Dublin, Dublin, Ireland; 14 Department of Biophysics, School of Medicine, Istanbul Medipol University, Istanbul, 34815 Türkiye; 15 Cognitive Neuroscience Center (CNC), Universidad de San Andrés, Buenos Aires, Argentina; 16 Department of Neurology, Barcelona Brain Health Center, Hospital del Mar, Barcelona, Spain; 17 Department of Medicine and Life Sciences, Universitat Pompeu Fabra, Barcelona, Spain; 18 Braingaze SL, Mataró, Spain; 19 Catalan Institution for Research and Advanced Studies (ICREA), Barcelona, Spain

**Keywords:** Eye vergence, Pupil response, Tau pathology, Oddball, AT(N) framework

## Abstract

The locus coeruleus (LC), the earliest site of tau accumulation in Alzheimer’s disease, directly influences pupillary and vergence oculomotor systems. Whether task-evoked oculomotor dynamics can non-invasively differentiate AT(N) profiles, A+T+ (biological Alzheimer’s disease) and A−T+ (suspected non-Alzheimer’s tauopathy), remains unexamined despite their differential impact on LC-mediated modulation and cortical network integrity. Thirty-eight MCI individuals (12 A−T+, 26 A+T+), classified using validated CSF biomarker thresholds, completed a visual oddball paradigm under binocular eye-tracking. Linear mixed-effects models examined profile × condition interactions across full time series and six trial-level features; participant-level associations were tested using Firth-penalized logistic regression to account for the small and imbalanced sample. Profiles did not differ in oculomotor magnitude but diverged in temporal organization. Profile × condition interactions were significant for vergence global slope, vergence time to peak, and pupillary time to peak (all p-Holm ≤ 0.048). Timing differences were condition-dependent: A−T+ showed later responses during distractor trials, while A+T+ showed the most delayed timing during target trials. Subject-level associations were significant for vergence and pupillary time to peak, with greater condition-dependent modulation predicting higher A+T+ probability (ORs = 0.43 and 0.35; both *p *< 0.05); vergence global slope delta did not reach significance at the subject level. A−T+ showed superior target detection accuracy (89.7% vs. 82.5%, *p *= 0.003). Cognitive vergence and pupillary dynamics showed condition-dependent patterns differentiating AT(N) biological profiles in this MCI sample, supporting portable, low-cost eye-tracking as a non-invasive complement to CSF-based characterization, pending confirmation in larger, independent cohorts.

**Supplementary information:**

The online version contains supplementary material available at https://doi.org/10.1007/s11357-026-02487-2.

## Introduction

Alzheimer’s disease (AD) is a neurodegenerative condition driven by the progressive accumulation of specific proteinopathies that can be detected and biologically staged during life through validated biomarkers [1]. The societal impact of AD is substantial: an estimated 57.4 million people are currently affected worldwide, a number projected to surpass 152.8 million by 2050 as populations age [2]. Crucially, the neuropathological cascade underlying AD unfolds years before cognitive symptoms become apparent [1], which has historically constrained opportunities for early research and therapeutic intervention. In response to these challenges, the 2018 NIA-AA Research Framework established a biological rather than syndromal definition of AD, organizing biomarkers into the AT(N) classification system according to pathological process: amyloid-β (A), fibrillar tau (T), and neurodegeneration (N) [1]. The recently revised 2024 Alzheimer’s Association criteria further refined this framework by differentiating Core 1 biomarkers (early-changing phosphorylated tau fragments (p-tau181, p-tau217, p-tau231) and amyloid markers) from Core 2 biomarkers reflecting tau aggregate burden, and by anchoring AD diagnosis to abnormality on Core 1 biomarkers [3]. Across both frameworks, the A+T+ profile biologically defines AD, characterized by reduced CSF Aβ42 and elevated phosphorylated tau, while the A−T+ profile, tau-positive in the absence of detectable amyloid pathology, represents a biologically distinct entity whose etiology may primarily reflect primary age-related tauopathy (PART) or other non-Alzheimer tauopathies [4, 5]. This distinction is a central concern in geroscience, where disentangling disease-specific neurodegeneration from processes that accompany typical brain aging remains an open challenge, underscoring the relevance of characterizing the functional correlates associated with each biological profile.

Both A+T+ and A−T+ profiles share the presence of tau-related pathology, and post-mortem evidence consistently identifies the locus coeruleus (LC) as the earliest site of tau accumulation in the AD pathological cascade, preceding cortical involvement by years [4, 6].

Located in the pontine brainstem, the LC constitutes the principal noradrenergic nucleus, with extensive projections to cortical and subcortical structures implicated in arousal, attentional regulation, and oculomotor control [7–9]. Through direct anatomical connections with the Edinger-Westphal nucleus, LC activity influences pupillary responses [7, 10]. Regarding vergence, the LC sends noradrenergic projections to the superior colliculus [11], a structure in which distinct neuronal populations encode and modulate vergence eye movements [12], suggesting an indirect pathway by which LC activity may influence vergence control. Pupil diameter serves as a well-validated psychophysiological index of LC-noradrenergic function across multiple cognitive paradigms [7, 10], while cognitive vergence responses correlate with attentional allocation and visual event-related potentials in parietal regions [13, 14]. Given the shared embryological origin of ocular and neural tissues [15], cognitive vergence and pupillary assessment during structured cognitive tasks may offer a non-invasive window onto the functional integrity of neural systems affected early by tau pathology in both biological profiles.

Prior work has documented cognitive vergence and pupillary alterations in individuals with MCI and AD during oddball paradigms, demonstrating that both signals are sensitive to attentional network dysfunction associated with cognitive decline [16] and that oculomotor responses during such tasks are significantly modulated by CSF biomarker levels, including Aβ42, p-tau181, and the Aβ42/p-tau ratio, independently of age, sex, and MMSE [17]. However, a critical question remains unaddressed: whether oculomotor signatures differ specifically between biological profiles, that is, whether the presence of amyloid co-pathology in A+T+ individuals generates a qualitatively distinct oculomotor pattern compared to A−T+ individuals. This distinction is biologically meaningful because A+T+ and A−T+ profiles differ not only in amyloid status but in the degree to which cortical network coordination may be compromised. In A+T+, amyloid accumulation preferentially affects default mode network (DMN) regions including the posterior cingulate cortex and precuneus [18, 19], potentially disrupting the anti-correlated relationship between task-positive and task-negative networks necessary for efficient attentional engagement [20]. A−T+ individuals, spared from this amyloid-driven network disruption, may retain relative functional resilience under high attentional demand. Consistent with this view, previous literature reported that A−T+ older adults exhibited significantly greater hippocampal volume, cortical thickness across AD-associated regions, and cerebral glucose metabolism compared to A+T+ individuals, while remaining indistinguishable from cognitively unimpaired healthy A−T− controls on all neurodegenerative markers [21], suggesting that tau protein in the absence of amyloid does not produce the same degree of structural and metabolic compromise. Whether these differences in pathological profile translate into detectable differences in task-evoked oculomotor dynamics has not been examined. A recent review of eye-tracking as a digital biomarker in neurodegenerative disease identified the lack of phenotypically well-characterized cohorts with biologically defined disease stages as a key limitation constraining the interpretability and comparability of existing oculomotor findings [22]. The present study directly addresses this gap by classifying participants according to AT(N) biological profiles using validated CSF biomarker thresholds.

This question carries direct clinical relevance. Profile characterization under both the 2018 NIA-AA framework and the revised 2024 criteria relies on CSF extraction or PET imaging, procedures that are invasive, costly, and poorly suited to population-level monitoring or routine longitudinal follow-up [18]. Although blood-based biomarkers such as plasma p-tau217 offer a less invasive alternative with increasing clinical availability, they share with CSF and PET the limitation of providing static indices of pathological burden without capturing how the brain responds functionally to cognitive demand. Thus, oculomotor assessment during structured attentional tasks could complement existing biomarker approaches by providing an accessible, non-invasive measure of functional network integrity, particularly informative in contexts where pathological burden and functional capacity may dissociate. Critically, the oculomotor assessment employed here requires only a calibrated remote eye-tracker and a standard laptop, imposes no consumables or procedural risk, and is completed within approximately 6 min, representing a substantially lower infrastructural burden than CSF extraction, PET imaging, or even blood-based assays requiring laboratory processing.

Building on these considerations, the present cross-sectional study aims to determine whether cognitive vergence and pupillary response patterns in individuals with MCI classified according to AT(N) biological profiles can functionally differentiate the A−T+ profile from the A+T+ profile. By establishing these relationships within the AT(N) framework, we seek to advance the validation of cognitive vergence and pupillary responses as accessible functional oculomotor signatures that complement CSF-based assessments by providing insight into the patient’s cerebral functionality, particularly the attentional and oculomotor networks, thus bridging biological and functional manifestations for early identification and monitoring of different neuropathological profiles.

## Methods

**Fig. 1:**
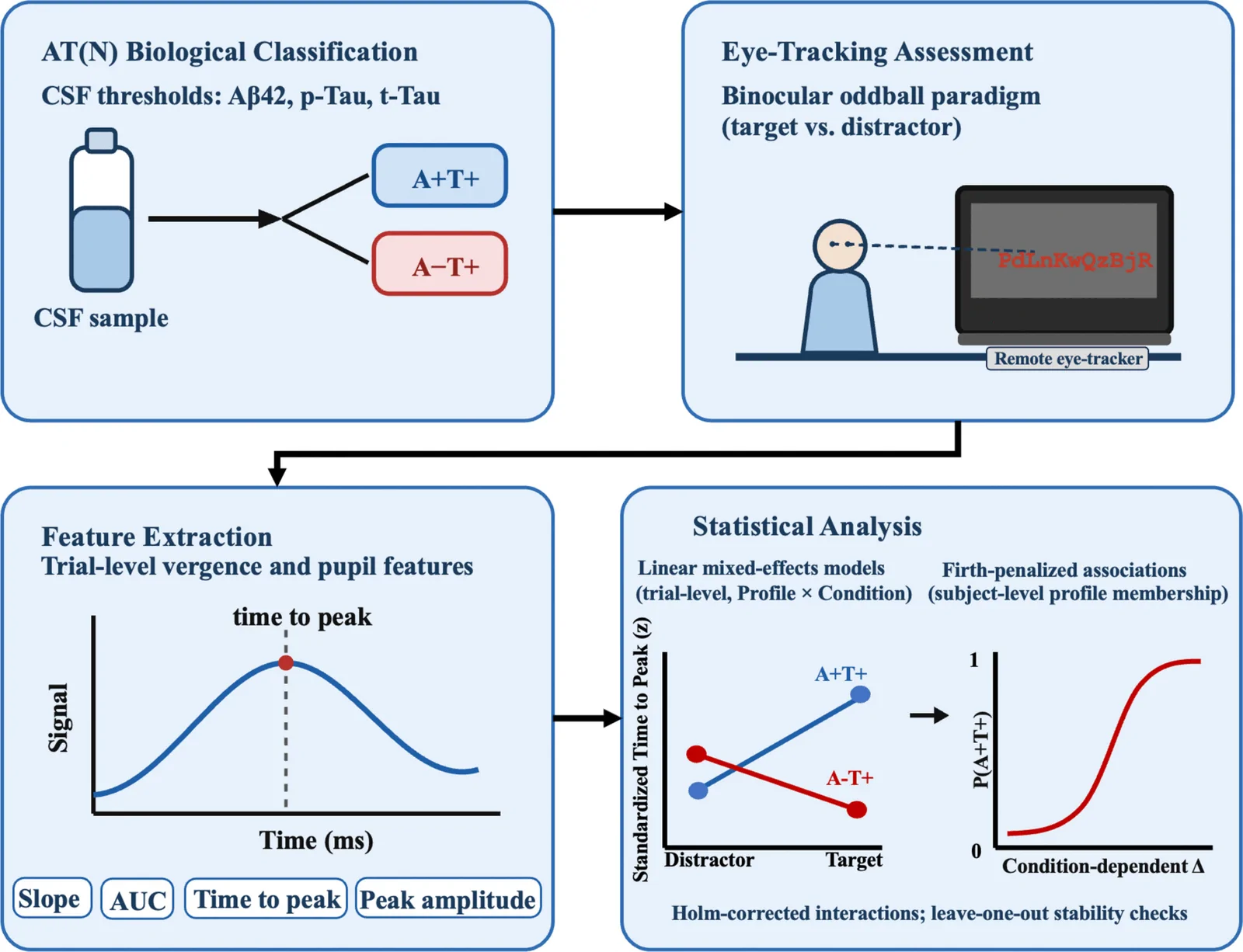
Overview of the study design. Participants are classified into AT(N) biological profiles (A+T+, A−T+) using validated CSF biomarker thresholds and complete a binocular oddball eye-tracking paradigm. Trial-level vergence and pupil features are extracted and analyzed using linear mixed-effects models (profile × condition) and Firth-penalized logistic regression to test profile-level associations

A cross-sectional design was used in this study. Ethical approval was obtained from the Ethics Committees of the University of Barcelona (HCB/2021/0668) and was conducted in accordance with the Declaration of Helsinki. The study followed the STROBE reporting guidelines for cross-sectional studies [23], and the corresponding checklist is provided as supplementary material. Figure 1 provides a schematic overview of the overall study design, from AT(N) biological classification to profile-level statistical association.

### Participants

Thirty-eight individuals in MCI stage (26 women, 12 men) from two Barcelona-based hospitals (Hospital del Mar and Hospital Clínic) participated in this study. All participants underwent lumbar puncture for CSF extraction as part of their clinical evaluation. Based on these clinical assessments, all participants showed abnormal CSF core AD biomarkers in accordance with Alzheimer’s Association criteria [3].

Written informed consent was obtained from all participants or their legal representatives prior to enrollment. Participants and caregivers received comprehensive verbal and written instructions regarding all experimental procedures.

### Biological profile classification

Biological profiles were established following the AT(N) framework [1] using population-specific CSF biomarker thresholds validated for Spanish cohorts. Hospital del Mar participants were classified using CORCOBIA study thresholds [24]: Aβ42 < 750 pg/mL defined amyloid positivity (A+), p-tau > 69.85 pg/mL indicated tau positivity (T+), and t-Tau > 522.0 pg/mL marked neurodegeneration (N+). For Hospital Clínic participants, institutional validated cutoffs were applied: Aβ42 ≤ 600 pg/mL (A+), p-tau > 65 pg/mL (T+), and t-Tau > 385 pg/mL (N+). Despite the use of center-specific thresholds, the same classification procedure and analytical approach were applied uniformly across participants. Hospital center was included as a covariate in all statistical models (see statistical analysis section); it did not reach statistical significance in any analysis, suggesting that center-related variability did not systematically influence the results.

Based on these cutoff points, participants were stratified into two primary biological profiles: the A+T+ profile, characterized by concurrent amyloid and phosphorylated tau abnormalities, and the A−T+ profile, defined by tau abnormalities in the absence of amyloid pathology.

Given the exploratory nature of this study and limited sample size (*n *= 38), our classification approach incorporated additional considerations. Two participants presenting A−T−(N)+ profiles (isolated elevation of total tau) were grouped with the A−T+ profile, justified by established evidence demonstrating total tau as a sensitive marker across various tauopathies and an important biomarker of neurofibrillary degeneration [5, 25].

To further assess the robustness of this decision, a sensitivity analysis was conducted excluding these two participants (see statistical analysis and supplementary material) and did not meaningfully alter the pattern of findings. This methodological approach aligns with the AT(N) framework’s explicit recommendation for adaptation “when it is fit for the purpose of specific research goals” [1, 3], maintaining consistency with NIA-AA principles while enabling meaningful biological stratification.

CSF concentrations of Aβ42, t-Tau, and p-tau were quantified using single molecule array technology (Lumipulse platform) following standardized institutional protocols [26] at each participant’s respective hospital.

### Sample size and statistical power

Given that participant enrollment was contingent on the availability of CSF biomarker data (requiring lumbar puncture as part of a separate clinical protocol) rather than guided by formal power calculations, we performed a post hoc simulation-based power analysis to evaluate the adequacy of our sample for detecting the effects of interest.

Standard power formulas are not appropriate for hierarchical time-series data, as they assume independence between observations. To account for the nested structure of our dataset (repeated temporal measurements within participants) and the random effects specification of our models (participant-level intercepts and time slopes), we used a parametric bootstrap approach, which is more appropriate than analytical formulas for mixed-effects models with this type of data structure [27]. Specifically, we generated 1000 new datasets by drawing from the estimated parameter distributions of the fitted mixed-effects model and refitting the full model to each simulated dataset with SIMR r package approach [28].

For the two primary oculomotor signals (cognitive vergence: *β* = 0.096, SE = 0.011, *t* = 8.99, *p *< 0.001; pupillary response: *β* =  − 0.060, SE = 0.010, *t* =  − 5.84, *p *< 0.001), post hoc power estimates at the observed sample size (*n *= 38) were 100% (95% CI, [98.1%, 100%]) for both signals, suggesting that the sample was adequate to detect effects of the magnitude observed. We further examined how this estimate varied across smaller hypothetical samples by repeatedly subsampling participants (*n *= 20, 25, 30, 35) and rerunning the simulation procedure. Both signals yielded power exceeding 90% across all evaluated sample sizes, indicating that effects of the observed magnitude would likely have been detectable even with a more modest sample.

### Inclusion/exclusion criteria

Participants were eligible for enrollment if they met the following criteria: (1) age ≥ 65 years; (2) availability of recent CSF biomarker analysis (Aβ42, p-tau, t-Tau) obtained through lumbar puncture as part of clinical evaluation; (3) adequate visual acuity (with or without corrective lenses) to perceive on-screen stimuli; and (4) capacity to provide informed consent or availability of a legal representative to provide consent on behalf of the participant.

Participants were excluded based on the following criteria: (1) severe cognitive impairment (Mini-Mental State Examination score < 10); (2) documented neurological conditions with clinically significant cognitive impact (e.g., cerebrovascular disease); (3) severe psychiatric diagnoses; (4) structural brain abnormalities identified through neuroimaging that could contribute to cognitive dysfunction (e.g., intracranial neoplasms); (5) ophthalmological conditions interfering with task performance (including blindness, strabismus, nystagmus, or significant retinal/oculomotor pathology); and (6) insufficient Spanish language comprehension.

### Eye-tracking equipment

Ocular data acquisition utilized the BGaze system (BraingazeSL, Mataró, Spain), a portable desktop-based platform that integrates stimulus presentation with binocular eye position and pupillometry recording on a standard laptop computer. Visual stimuli were displayed at 1024 × 768-pixel resolution. Eye movements were captured using a Tobii 5L remote eye tracker (Tobii Technology AB, Sweden) mounted below the screen, sampling at 33 Hz without requiring physical contact with the participant. Manufacturer specifications report spatial accuracy of 0.4° visual angle and precision of 0.32° visual angle under optimal binocular viewing conditions. The 33-Hz sampling rate provides a temporal resolution of approximately 30 ms per sample. Although this is below rates used for high-frequency oculomotor metrics such as saccade kinematics, cognitive vergence and pupillary responses are slow signals whose discriminative features unfold over windows of 500–2000 ms post-stimulus. Even for the shortest extraction window (initial slope, 0–500 ms), this rate yields approximately 16 sampling points, sufficient for robust linear regression estimation. This is consistent with recommendations for cognitive pupillometry [29] and with prior work employing the same system in MCI and AD populations [16, 17].

### Procedure

Testing occurred in a dedicated room within the hospital facility under controlled lighting conditions (lights extinguished, curtains closed to achieve dim ambient illumination 50 lx on average). Participants were positioned approximately 50 cm from the stimulus display screen, with the eye-tracker mounted directly below. Corrective lenses were permitted. Head position was stabilized using a chinrest to minimize motion artifact.

### Task design

**Fig. 2:**
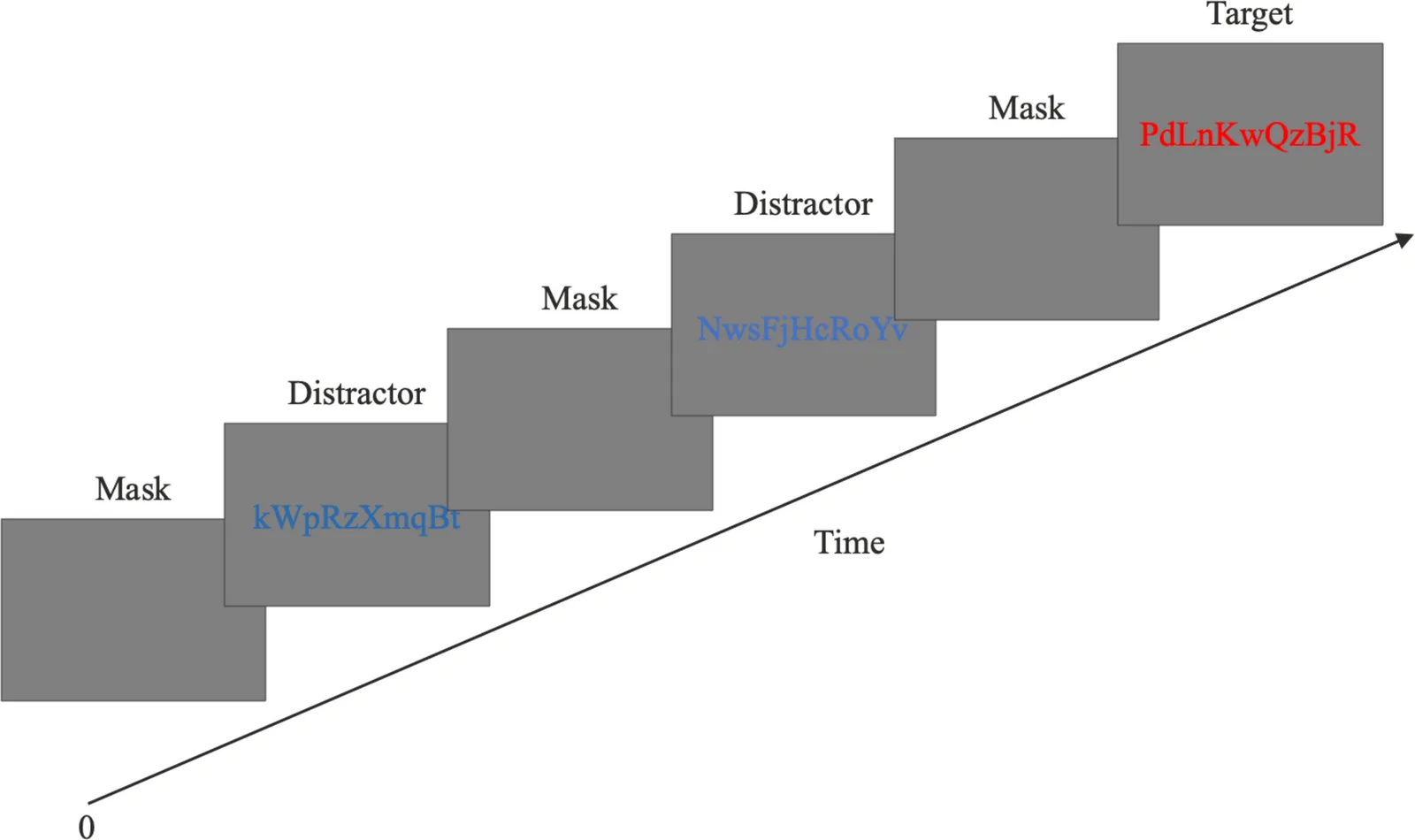
Oddball task sequence. Strings of symbols are presented for 2 s, followed by a gray mask. In 80% of the trials, the symbols were printed in blue, and in the remaining 20% they were printed in red

We employed a visual oddball paradigm, selected for its robust activation of distributed brain networks including the locus coeruleus [30]. The task comprised 120 discrete trials, each beginning with a 2000-ms gray mask screen, followed by 2000 ms presentation of a centrally positioned letter string (Fig. 2).

Each stimulus consisted of 11 randomly generated characters in mixed upper and lower case, arranged to avoid forming meaningful words or recognizable acronyms that might introduce cognitive bias. Stimuli were differentiated solely by font color: 80% appeared in blue (frequent/non-target condition) while 20% appeared in red (rare/target condition). Participants received instructions to maintain visual fixation on the screen and execute a button press exclusively upon detecting red-colored strings. Verbal instruction reinforcement was provided by the experimenter as needed during task performance.

Following standard oddball nomenclature, red letter strings constituted target stimuli while blue strings served as distractor stimuli. Stimulus presentation order was pseudorandomized. Continuous binocular eye-tracking was maintained throughout the ~ 6-min task duration. Prior to task initiation, a five-point binocular calibration procedure was completed for each participant to ensure measurement accuracy.

### Signal preprocessing and functional oculomotor signal extraction

Cognitive vergence and pupil responses were extracted during the attentional task as oculomotor signature. Eye-tracking recordings underwent systematic preprocessing to ensure signal integrity. Quality control procedures excluded trials containing fixations outside the display area (normalized coordinates < 0 or > 1) and physiologically implausible vergence angles (< 0° or > 20°). Trials were retained only if valid data comprised at least 30% of the recording period, ensuring adequate signal-to-noise ratios for subsequent analyses. After excluding trials with more than 30% missing samples, the remaining trials showed limited need for reconstruction: 71.8% required ≤ 20% interpolation and 28.2% required < 5%, consistent with expected blink-related signal interruptions in a clinical population with established eye-tracking methodologies [29].

Cognitive vergence angle was computed using a vector-based geometric approach from three-dimensional eye position coordinates. Two-dimensional gaze coordinates were first converted to physical dimensions (millimeters) using the monitor’s specifications (340 mm × 190 mm). Gaze direction vectors were constructed from each eye’s spatial position to its corresponding point of regard on the screen. Vergence angle (*θ*) at each time point was calculated as the angle between left and right gaze vectors using vector dot product geometry, expressed in degrees thus relative vergence changes were computed as *υ*(*t*) = (*γ*(*t*) − *γ*_0_), where *γ*_0_ represents baseline vergence, this method was previously published by our team [14]. For pupil responses, absolute diameter changes were calculated as *π*(*t*) = *p*(*t*) − *p*_0_, where *p*(*t*) represents the mean of left and right pupil diameters. Both baseline values (*γ*_0_ and *p*_0_) were calculated as the mean of the first 7 temporal points (≈212 ms pre-stimulus period) following previous recommendations [29]. Both cognitive vergence and pupil signals were subjected to Gaussian smoothing using moving average filter prior to baseline correction to optimize signal-to-noise ratio.

To quantify the functional characteristics of oculomotor responses beyond simple amplitude measures, six features were extracted from each individual trial’s vergence and pupil response curves. Initial slope was computed over the first 500 ms, capturing the primary response dynamics. Global slope characterized the overall rate of change across the entire trial duration. Late slope was computed over 1000–2000 ms, reflecting sustained response or plateau phase. Temporal windows for slope extraction were determined through visual inspection of mean response curves. Linear regression was employed for slope estimation as it provides a robust single-parameter summary of the dominant directional trend within each temporal window, minimizing the influence of high-frequency noise while capturing the primary rate of signal change. Area under the curve (AUC) was computed using trapezoidal integration, the standard approach in pupillometry [29], representing the cumulative magnitude of the response. Time to peak identified the latency to maximum response amplitude. Peak amplitude measured the maximum value attained during the trial. Slope values were converted from milliseconds to seconds (multiplied by 1000) for interpretability. These features were extracted separately for both cognitive vergence and pupil responses, yielding 12 total features per trial (6 for each measure). All features were subsequently standardized (*z*-scores) prior to statistical modeling to enable direct comparison across measures with different units and scales. All the preprocessing and feature extractions were conducted in Python 3.x.

### Statical analysis

Data normality was assessed using Shapiro-Wilk tests and visual inspection of histograms and Q-Q plots. Pupil data were normally distributed; cognitive vergence data appeared approximately normal in histograms but showed slight tail deviations in Q-Q plots. Given the repeated-measures structure and large sample size (237,600 data points; 6600 per participant), linear mixed-effects models (LMMs) were used, as they are robust to minor normality deviations in large samples [31]. Center was initially evaluated as a fixed covariate but showed no significant effects across model specifications and was not retained in final models.

Analyses followed a hierarchical strategy operating at three levels of temporal aggregation. First, full cognitive vergence and pupil time series (*Z*-scored) were modeled using LMMs with random intercepts for participant and participant-specific random slopes for timestamp, capturing individual temporal dynamics. Since each signal constituted a single inferential test, type I error was controlled through a maximal random effects structure rather than multiple comparison correction [32]. Second, oculomotor signals were decomposed into trial-level features reflecting temporal dynamics (slope-based features), response timing, and response magnitude, each modeled separately using LMMs with random intercepts for participant. Holm correction was applied to profile × condition interaction terms within theoretically defined feature families, as these interactions represented the primary confirmatory hypotheses. Main effects of profile and condition were not corrected, serving as interpretive context. Third, features showing significant profile × condition interactions were aggregated at the participant level and entered into separate penalized binomial logistic regression models (Firth’s method) predicting biological profile membership [33]. Rather than entering condition as a within-subject factor, a delta score was computed for each participant as the difference between target and distractor condition means (Δ = Target − Distractor), reducing each model to a single predictor. This aggregation reduces model complexity and increases effective degrees of freedom, which is particularly advantageous given the small sample size. Firth’s approach was selected given the small and imbalanced sample, where maximum likelihood estimation produces biased coefficients. No multiple comparison correction was applied at this stage, as the three models were pre-selected based on the prior interaction tests and thus constituted a family of directed hypotheses rather than an exploratory screen. These models were designed to test pre-specified associations between condition-dependent oculomotor timing and biological profile membership rather than to develop or validate a diagnostic classifier, as the present sample falls below established minimum requirements for reliably estimating discrimination metrics such as AUC, sensitivity, and specificity [34]. To evaluate the stability of the two associations reaching subject-level significance, a leave-one-participant-out (LOSO) sensitivity analysis was performed, iteratively refitting each Firth model after excluding one participant at a time and evaluating the consistency of coefficient sign and significance across all 38 refits.

For LMMs, dependent variables were *Z*-scored prior to analysis; regression coefficients therefore reflect changes in standard deviation units of the oculomotor signal and are reported as standardized effect size estimates. For logistic regression models, odds ratios (OR) with 95% profile likelihood confidence intervals are reported as effect size indices.

Covariate effects of age, sex, and MMSE were evaluated in a sensitivity analysis (Supplementary Material). Covariates reached significance only for vergence and pupil peak power features and did so exclusively in main effects, with no significant covariate × interaction terms. Following the principle of parsimony, covariates were not retained in final models. A second sensitivity analysis excluded two A−T+ participants classified on the basis of total tau rather than p-tau181 values. All three analytical levels were re-estimated in this restricted sample. The only change in inferential outcome was the loss of significance for cognitive vergence time to peak (primary analysis: *p *= 0.048; restricted sample: *p *= 0.133), a feature already proximal to the significance threshold. No significant effect changed direction, and no non-significant effect crossed the *α* = 0.05 boundary in either direction, suggesting the attenuation reflects power loss rather than undue influence of those two participants. All remaining significant effects were maintained below *α* = 0.05. Significance threshold was set at *p *< 0.05 for all analyses. Analysis code is openly available [35], and all analyses were conducted in R (version 4.x).

## Results

The two biological profiles were well-matched on demographic and cognitive characteristics, with no significant differences in age, sex distribution, or general cognitive performance (Table 1). As expected by design, the groups diverged markedly on Aβ42 and Aβ42/p-tau ratio, confirming successful stratification of participants into biologically distinct profiles.

**Table 1:** Participants characterization

Variable	All *n *= 38	A−T+ *n *= 12	A+T+ *n *= 26	*p*-value (A−T+ vs. A+T+)
Age (y)	72.9 ± 5.83	73.4 ± 4.93	72.7 ± 6.28	0.975
Sex (W/M)	26/12	9/3	17/9	0.559
MMSE	22.4 ± 4.04	23.8 ± 3.93	21.7 ± 4.00	0.154
Aβ42	623 ± 281	946 ± 255	473 ± 123	** < 0.001**
p-tau181	160 ± 196	97.6 ± 70.7	131 ± 84.7	0.120
t-Tau	762 ± 370	754 ± 273	824 ± 373	0.172
Aβ42/p-tau ratio	7.80 ± 7.77	15.1 ± 10.6	4.6 ± 2.21	** < 0.001**

Continuous variables are presented as mean ± standard deviation; the categorical variable (sex) is presented as frequency. Group comparisons used independent-samples *t*-tests for continuous variables and the chi-square test for sex distribution. Values in bold indicate statistical significance (*p *< 0.05). *y* years, *W/M* women/men

### Functional oculomotor signatures: cognitive vergence and pupil response

**Fig. 3:**
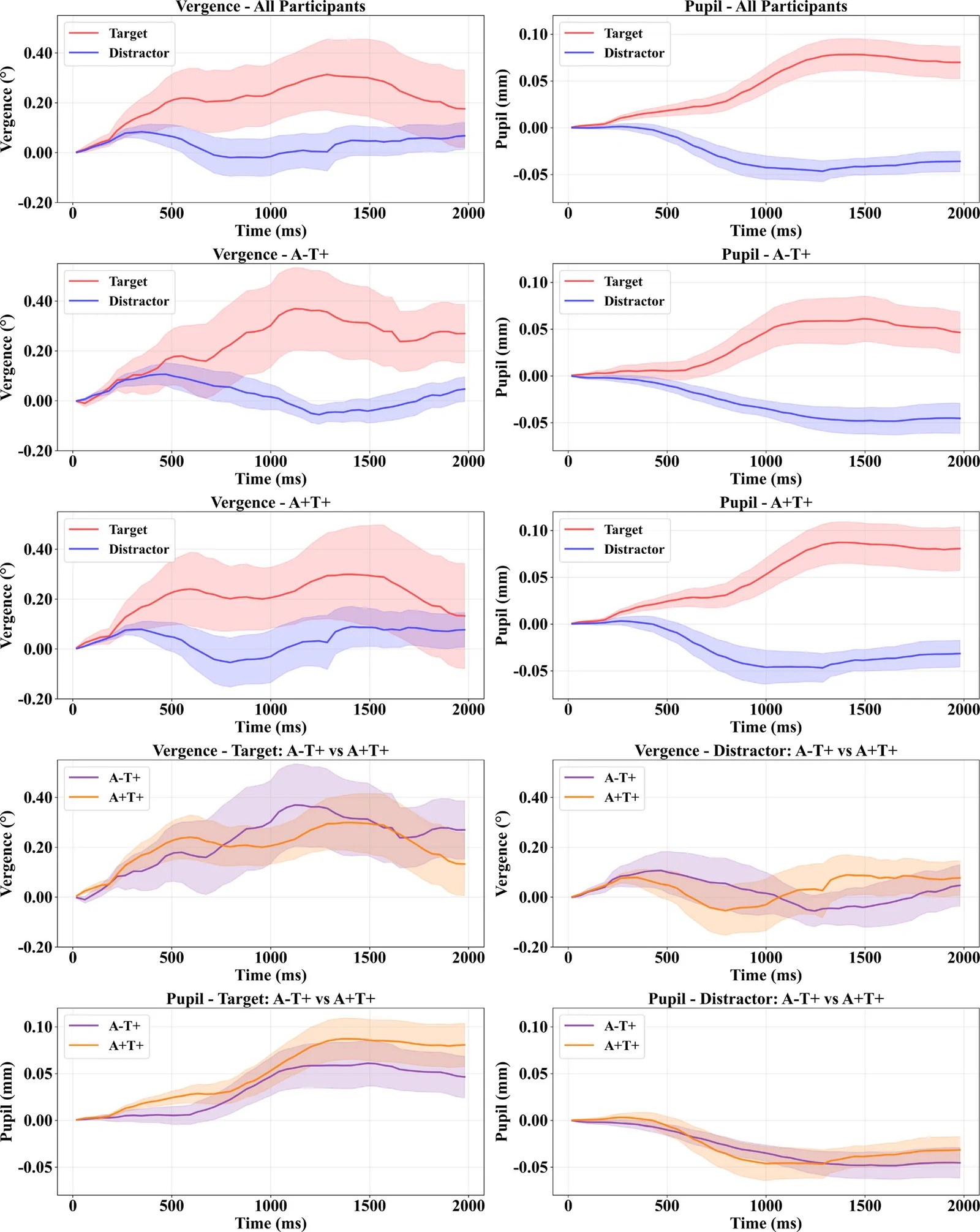
Cognitive vergence and pupil response during oddball task. Rows 1–2: average cognitive vergence and pupil responses during oddball target/distractor conditions according to biological profile. Rows 3–4: target vs. distractor comparisons by biological profile. Shaded areas, standard error; time, milliseconds from stimulus onset

Both cognitive vergence and pupillary signals showed systematic modulation by stimulus type across the full sample, with target stimuli consistently eliciting larger responses than distractors (Fig. 3). Neither cognitive vergence nor pupillary amplitude differed between profiles when averaged across conditions. However, significant interactions between stimulus type and biological profile emerged for both signals (Table 2), indicating that the magnitude of condition-dependent modulation varied between the two groups.

**Table 2:** Global and temporal analysis of cognitive vergence and pupil

Signal	Effect	*β*	95% CI	*p*
Cognitive vergence	Condition	0.039	[0.028, 0.050]	** < 0.001**
	Profile	−0.053	[− 0.219, 0.112]	0.515
	Interaction	0.096	[0.075, 0.117]	** < 0.001**
Pupil	Condition	0.467	[0.456, 0.477]	** < 0.001**
	Profile	−0.045	[− 0.211, 0.121]	0.587
	Interaction	−0.060	[− 0.080, − 0.040]	** < 0.001**

Reference categories: condition = distractor, profile = A+T+. Bold values indicate *p *< 0.05. *β* standardized regression coefficient, *CI* confidence interval

The pattern of this modulation differed between the two oculomotor systems. For cognitive vergence, the A−T+ profile showed greater condition-dependent differentiation between target and distractor stimuli compared to the A+T+ profile. For pupillary responses, the opposite pattern was observed: A+T+ showed greater condition-dependent differentiation than A−T+. These divergent interaction patterns suggest that the two biological profiles were associated with distinct oculomotor dynamics depending on stimulus type, with each profile showing differential condition-sensitivity across cognitive vergence and pupillary systems.

### Accuracy *response*

**Fig. 4:**
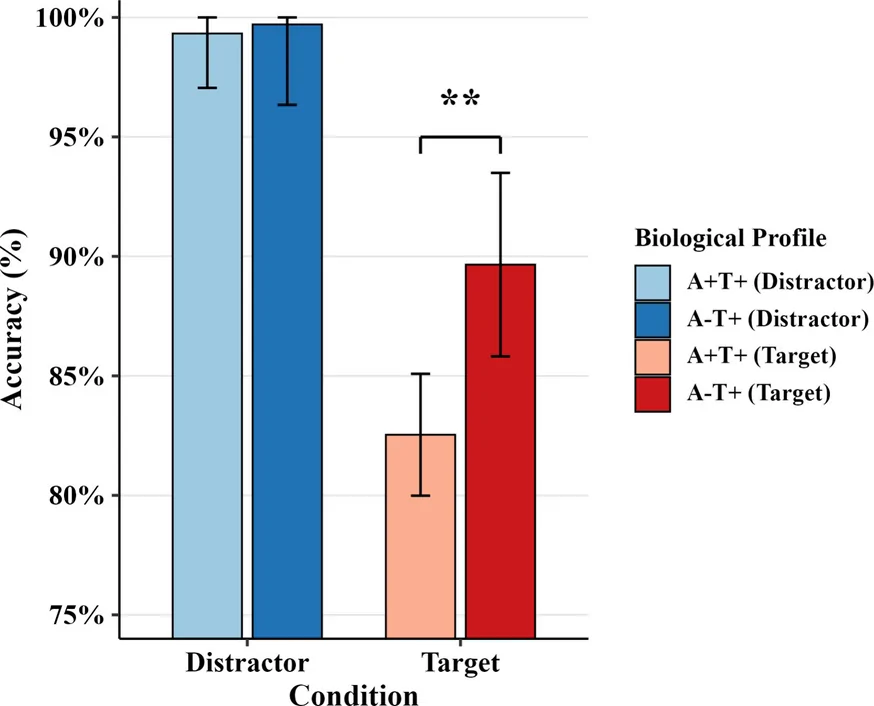
Accuracy comparison to oddball task. Accuracy in percentage, an asterisk indicates significant difference between profiles

Behavioral performance revealed profile-dependent differences that varied by stimulus condition (Fig. 4). Both groups performed at comparable levels on distractor trials, with near-ceiling accuracy (A+T+: 99.3%, 95% CI [97.1%, 100.0%]; A−T+: 99.7%, 95% CI [96.3%, 100.0%]; *β* = 0.004, 95% CI [− 0.037, 0.044], *p *= 0.851). Performance diverged on target trials, where A−T+ maintained significantly higher detection accuracy than A+T+ (A+T+: 82.5%, 95% CI [80.0%, 85.1%]; A−T+: 89.7%, 95% CI [85.8%, 93.5%]; *β* = 0.071, 95% CI [0.025, 0.117], *p *= 0.003), as modeled using a linear mixed-effects model with profile and condition as fixed effects and participant as random intercept.

### Functional oculomotor signatures: trial-level features

Trial-level feature analysis revealed condition-dependent oculomotor patterns associated with biological profile (Table 3). Linear mixed-effects models identified significant profile × condition interactions for vergence global slope, vergence time to peak, and pupillary time to peak. For vergence global slope, the two profiles did not differ during distractor trials but diverged during target trials, where A−T+ showed steeper vergence dynamics than A+T+. For vergence time to peak, A−T+ showed later peak latencies than A+T+ during distractor trials; this pattern reversed during target trials, where A+T+ exhibited the slower response. Pupillary time to peak followed the same directional pattern, with A−T+ exhibiting later peak latencies than A+T+ during distractor trials and this difference reversing during target trials, where A+T+ showed the delayed response.

**Table 3:** Condition-dependent temporal oculomotor features differentiating AT(N) biological profiles

Feature	Effect	*β*	95% CI	*p*	p-Holm
Linear mixed-effects models					
Cognitive vergence					
Slope global	Condition	−0.025	[− 0.115, 0.065]	0.586	-
	Profile	−0.083	[− 0.250, 0.084]	0.320	-
	Interaction	0.206	[0.035, 0.377]	**0.018**	**0.036**
Time to peak	Condition	0.260	[0.170, 0.350]	** < 0.001**	**-**
	Profile	0.095	[− 0.026, 0.216]	0.140	-
	Interaction	−0.174	[− 0.331, − 0.017]	**0.048**	**0.048**
Pupil					
Time to peak	Condition	0.282	[0.192, 0.372]	** < 0.001**	**-**
	Profile	0.107	[− 0.037, 0.248]	0.136	**-**
	Interaction	−0.253	[− 0.422, − 0.079]	**0.003**	**0.007**
Binary logistic regression					
Cognitive vergence		Log-odds	OR [95% CI]		
Slope global	Delta-z	0.217	1.24 [0.66; 2.38]	0.471	
Time to peak	Delta-z	− 0.831	0.43 [0.19; 0.99]	**0.049**	
Pupil					
Time to peak	Delta-z	− 1.045	0.35 [0.11; 0.88]	**0.023**	

Reference categories: condition = distractor; profile = A+T+. For mixed-effects models: *β* standardized coefficient in SD units of the oculomotor signal, *CI* confidence interval, *p-Holm* Holm-corrected *p*-value, dashes indicate main effects not subject to correction. For logistic regression models: *β*, log-odds; *OR*, odds ratio with 95% profile likelihood confidence interval; the predictor is the delta score (Δ = Target − Distractor, *Z*-scored); OR > 1 indicates higher probability of A−T+ profile membership. No multiple comparison correction was applied to logistic regression models, as the three models were pre-selected based on prior significant interaction terms. Bold = *p *< 0.05

Binary logistic regression was consistent with this condition-dependency extended to the classification of biological profile. At the subject level, participant-averaged delta scores (Δ = Target − Distractor, *Z*-scored) for each of the three features were entered into separate Firth-penalized logistic regression models. Vergence global slope delta did not significantly predict profile membership, suggesting that condition-dependent slope differences, while present at the trial level, were insufficiently stable within individuals to support subject-level discrimination. Vergence time to peak delta was associated with profile membership, with greater condition-dependent modulation of vergence peak latency associated with higher probability of A+T+ profile membership. Pupillary time to peak delta showed the same pattern with a larger effect magnitude, with greater condition-dependent modulation of pupillary peak latency similarly associated with higher probability of A+T+ profile membership.

To further evaluate the stability of these two significant subject-level associations, a LOSO sensitivity analysis was performed, refitting each Firth model 38 times while excluding one participant at a time. The direction of the association was retained in all 38 refits for both vergence time to peak (log-odds range, −1.627 to −0.635) and pupillary time to peak (log-odds range, −1.369 to −0.802), indicating that neither association reversed sign upon exclusion of any single participant. Statistical significance was highly stable for pupillary time to peak, remaining below *p *= 0.05 in 37 of 38 refits (*p* range, 0.008–0.064), but considerably more fragile for vergence time to peak, remaining significant in only 8 of 38 refits (*p* range, 0.006–0.172), consistent with its *p*-value in the primary analysis (*p *= 0.049) lying close to the significance threshold. Notably, the magnitude of the vergence time to peak association remained substantial across all refits despite this fluctuation in significance, indicating that at this sample size statistical significance, rather than effect size, was the quantity most sensitive to the exclusion of individual participants [36].

## Discussion

This study aimed to determine whether cognitive vergence and pupillary response patterns in individuals with MCI classified according to AT(N) biological profiles can functionally differentiate the A−T+ profile from the A+T+ profile. The present findings suggest that the two profiles are not distinguished by the overall magnitude of their oculomotor responses, but rather by the temporal organization of those responses, specifically by when peak responses occur, and that this temporal differentiation is condition-dependent, varying systematically between distractor and target trials. Critically, the pattern of differentiation reflects differences in temporal organization rather than overall response speed: A−T+ individuals showed relatively earlier peak latencies during target trials, whereas A+T+ individuals showed the most delayed peak latencies under the same conditions. This pattern was observed across analytical levels, from continuous signal dynamics to trial-level feature decomposition and subject-level analyses, lending convergent support to the main observation.

### Condition-dependent temporal dissociation between biological profiles

The condition-dependent nature of the observed dissociation is central to interpreting these findings. Both profiles showed comparable oculomotor response magnitudes overall, yet their timing signatures diverged in a direction that depended on whether the stimulus was a distractor or a target. For both cognitive vergence and pupillary time to peak, A−T+ showed later responses under distractor conditions, while A+T+ showed the most delayed timing under target conditions. This reversal implies that the two profiles differ not in the degree of oculomotor slowing but in the conditions under which slowing is expressed, suggesting that qualitatively distinct neural processes may underlie the timing differences in each profile. The behavioral findings parallel this pattern: both groups performed comparably on distractor trials, where accuracy was near ceiling, while A−T+ maintained higher detection accuracy than A+T+ specifically on target trials. This convergence between behavioral and oculomotor condition-dependency strengthens the interpretation that the observed differences are functionally meaningful rather than incidental variability.

### Neural mechanisms underlying profile-specific oculomotor timing

The mechanistic account below is necessarily interpretive, as LC integrity, DMN connectivity, and network-level compensation were not directly measured in this study.

These results are consistent with the differential impact that isolated tau versus combined amyloid-tau pathology has on the neural systems that govern the temporal dynamics of attentional processing. Both profiles carry tau pathology, and post-mortem findings place the LC as the earliest structure affected by tau in the AD cascade, years before cortical involvement [4, 6]; through noradrenergic projections to the superior colliculus and Edinger-Westphal nucleus, the LC governs arousal, attention, and oculomotor output [7, 9]. Critically, the LC operates in two functionally distinct modes: tonic firing that regulates baseline attentional state and phasic bursting that is selectively evoked by salient stimuli such as oddball targets [37]. Pupil diameter has been established as a reliable psychophysiological index of LC activity in both its tonic and phasic modes, with trial-level associations between pupillary responses and LC neural activity demonstrated in oddball paradigms specifically [10, 30, 37]. Under distractor conditions, which impose minimal attentional demand and engage primarily tonic noradrenergic regulation, the globally delayed pupillary time to peak observed in the A−T+ group may reflect subtle alterations in tonic LC-mediated modulation associated with tau burden at this structure. Tau pathology in the LC is known to disrupt both tonic and phasic noradrenergic output; early LC tau burden shifts neuronal activity toward a state of persistent high tonic discharge that secondarily impairs the efficiency of phasic bursting [38]. Under this tonic-phasic imbalance, such differences in tonic regulation would be most detectable under low-demand conditions, where LC tonic output constitutes the primary driver of attentional state and compensatory network recruitment is not available to mask the underlying dysfunction [30, 39].

Under target conditions, successful oculomotor modulation requires more than tonic LC regulation. Oddball target processing robustly engages distributed attentional networks: the dorsal attention network (top-down selection), the ventral attention network (bottom-up reorientation to salient stimuli), the frontoparietal network (executive control and decision-related processing), and the hippocampus (attention-dependent memory encoding) [30, 40–42]. The phasic LC response to target stimuli has been specifically linked to the P300 event-related potential and to the encoding of salience information through gamma power increases in prefrontal cortex [30, 37, 40]. Efficient engagement of this network also requires adequate DMN suppression. Because amyloid preferentially targets DMN hubs such as the posterior cingulate cortex and precuneus [19, 43], and is linked to abnormal DMN connectivity even before cognitive symptoms emerge [19, 20, 43], impaired suppression of this network during goal-directed processing can disrupt the anti-correlation between task-positive and task-negative systems that efficient attention depends on [20, 30, 44]. In A+T+, where amyloid pathology is superimposed on tau burden, this additional disruption of cortical network coordination may be associated with the most delayed oculomotor timing observed during target processing in this profile. The integrity of frontoparietal connectivity has been specifically linked to attentional resilience in the context of early LC tau pathology, with higher left frontoparietal network connectivity attenuating the negative effects of LC tau burden on cognitive performance [45]. In A−T+, where amyloid disruption is absent, this network-level resilience may be better preserved, which would be consistent with the observed maintenance of target detection accuracy and the absence of abnormally delayed target-related timing in this profile. This interpretation is structurally supported by evidence that, unlike A+T+, A−T+ older adults show hippocampal volume and AD-region cortical thickness comparable to cognitively unimpaired controls [21], suggesting the oculomotor timing advantage observed here during target processing reflects a preserved neural substrate rather than mere compensatory recruitment.

It is also important to consider that the A−T+ profile, as classified in this study, does not represent a single homogeneous etiological entity. Elevated tau in the absence of amyloid pathology can arise from PART, a neuropathologically defined entity that shares tau positivity with AD but follows a distinct anatomical progression largely restricted to medial temporal structures [46], as well as from other non-Alzheimer tauopathies with distinct underlying mechanisms. The present classification approach, based on validated CSF thresholds, cannot further distinguish among these etiologies within the A−T+ group. This biological heterogeneity is a defining feature of the A−T+ profile itself rather than a shortcoming of the present design, and it likely contributes variability to the oculomotor timing patterns observed within this group, potentially diluting or, depending on subgroup composition, sharpening the group-level differences reported here relative to A+T+. Future work incorporating tau-PET or additional neuropathological characterization could determine whether the oculomotor timing advantage observed in A−T+ during target processing is uniform across these underlying etiologies or specific to a subset, such as PART. The inclusion of two participants classified as A−T−(N)+ within the A−T+ group in the present sample reflects this same underlying heterogeneity rather than a separate methodological limitation, and the sensitivity analysis reported in the “Selectivity and robustness of oculomotor timing features” section, which excluded these two participants, indicated minimal impact on the pattern of findings.

### Selectivity and robustness of oculomotor timing features

Among all features extracted, only timing metrics (time to peak for both cognitive vergence and pupil) consistently differentiated the profiles across both trial-level and subject-level analyses. Slope-based metrics capturing the rate of signal change showed more limited and less stable discrimination. This selectivity is consistent with prior evidence that peak latencies of both cognitive vergence and pupil responses are sensitive to attentional network dysfunction in cognitive impairment [16], and further extends this observation by showing that even within a cognitively impaired sample, timing features may carry information about the specific molecular pathology combination present, a pattern that will require replication in larger cohorts to establish its reliability. Time to peak integrates information across the full trial window, reflecting the cumulative temporal unfolding of the response rather than summarizing a single phase, which may account for its greater robustness [47]. The selective failure of slope metrics at the subject level, despite their significance at the trial level for cognitive vergence global slope, suggests that condition-dependent slope differences are real but insufficiently stable within individuals to serve as reliable subject-level markers, whereas timing metrics appear to capture more stable individual-level characteristics of temporal processing.

The differential robustness of cognitive vergence and pupillary time to peak also warrants comment. Both timing features showed significant effects across analytical levels, with pupillary time to peak showing greater effect magnitude than cognitive vergence time to peak. This difference in sensitivity is broadly consistent with the anatomical organization of these systems: pupillary modulation is more directly coupled to LC activity through both sympathetic and parasympathetic pathways and has been validated as a psychophysiological index of LC-noradrenergic function across multiple paradigms [7, 10, 39], while vergence responses depend additionally on circuits involved in spatial attention and binocular processing that introduce condition-specific influences [9, 14, 16]. These systems are not independent, however: vergence movements are known to trigger pupillary responses through direct anatomical coupling at the Edinger-Westphal nucleus, which receives input from vergence-related circuits and coordinates both oculomotor outputs [7–9]. Under this coupling, cognitive vergence responses temporally precede and may condition the subsequent pupillary response, such that the greater robustness of pupillary timing across conditions could partly reflect the integration of vergence-driven input with direct LC-noradrenergic modulation at the Edinger-Westphal level [7, 9]. The fact that LC neurons phase-lock to prefrontal and hippocampal oscillatory rhythms that synchronize to behavioral events [8, 41, 42] further supports the idea that pupillary timing captures a more direct and condition-independent readout of noradrenergic state, whereas vergence timing may reflect additional demand-dependent processes that emerge under attentional conditions.

The sensitivity analysis excluding the two participants classified on the basis of total tau rather than p-tau181 provided general support for the robustness of the findings. All effects that were significant in the primary analysis maintained the same direction in the restricted sample, and no non-significant effect crossed the significance threshold in either direction. The only change in inferential outcome was the loss of significance for cognitive vergence time to peak, a feature already proximal to the significance threshold in the primary analysis. This attenuation is consistent with reduced statistical power in the smaller sample rather than undue influence of those two participants, given the absence of directional reversals across the full set of effects. This supports interpretability within the AT(N) framework as applied, while acknowledging that measurement heterogeneity within the A−T+ group warrants attention in future work with larger samples.

### Clinical implications

A distinctive feature of the present findings is that cognitive vergence and pupillary temporal dynamics appear to reflect not merely which molecular pathology profile is present, but the functional state of the neural systems that profile compromises. The condition-dependent pattern of oculomotor timing differences captures how each profile processes attentional demands differently, providing a dynamic readout of brain network integrity rather than a static proxy for pathological load. This distinction is clinically relevant: current AT(N) profile characterization relies on CSF extraction or PET imaging, procedures that are invasive, costly, and difficult to implement at scale for population monitoring or longitudinal follow-up [18]. Although blood-based biomarkers such as p-tau217 represent a less invasive alternative that is increasingly available in clinical settings and shows excellent diagnostic performance for amyloid and tau status [48], they share with CSF and PET the limitation of providing a static index of pathological burden without capturing how the brain responds functionally to cognitive demand, and their broader clinical implementation still faces open translational challenges [49]. Oculomotor assessment during structured attentional tasks could complement these approaches precisely in this regard, offering an accessible, non-invasive measure of functional brain integrity that may be particularly informative in contexts where pathological burden and functional capacity dissociate [15, 50]. We envision this approach primarily as a functional complement to these molecular biomarkers rather than a replacement for them, with a secondary role as a screening tool in resource-constrained settings, discussed below; its value for longitudinal monitoring of disease progression remains an open question to be addressed in future longitudinal cohorts. Beyond blood-based biomarkers, this functional signature may also complement structural neuroimaging and digital cognitive assessments, which are increasingly combined into staged diagnostic workflows in primary care settings [51]. While the magnitude of the profile-dependent timing differences reported here was statistically robust, whether these effect sizes correspond to a clinically meaningful threshold remains an open question, one that awaits validation in larger, better-powered cohorts with established benchmarks for the clinical interpretation of oculomotor timing metrics [22].

From a point-of-care perspective, the technical requirements of the present paradigm are notably modest. The assessment was administered using a portable remote eye-tracker and a standard computer, without consumables, physical contact, or specialized clinical infrastructure. The task duration of approximately 6 min is compatible with routine clinical appointments. These characteristics position cognitive vergence and pupillary assessment as a candidate screening tool for settings where access to CSF-based or neuroimaging diagnostics is constrained by cost, equipment availability, or procedural invasiveness, including primary care contexts and healthcare systems with limited specialist resources. Within neurological practice, functionally distinguishing A−T+ from A+T+ profiles could inform clinical decision-making, for instance supporting more conservative monitoring strategies in A−T+ individuals while prioritizing closer follow-up or eligibility assessment for amyloid-targeted therapies in A+T+ patients.

The observation that A−T+ profiles maintained better target detection accuracy and relatively preserved oculomotor timing under high-demand conditions, despite carrying tau pathology, is consistent with evidence that molecular pathology alone does not determine functional outcomes and that network-level compensatory capacity plays an important role during early disease stages [45, 50]. This raises the possibility that temporal oculomotor metrics could serve as accessible indicators of functional reserve and may be particularly relevant for monitoring treatment response in interventions targeting noradrenergic function or attentional network synchronization, where changes in functional capacity may precede detectable changes in static biomarkers [52]. The differential oculomotor signatures between profiles also suggest that these populations may respond differently to network-targeted interventions, a question that warrants direct investigation in future trials.

Taken together, these findings indicate that condition-dependent timing, rather than magnitude, of cognitive vergence and pupillary responses distinguishes AT(N) biological profiles in this MCI sample, positioning portable eye-tracking as a promising digital biomarker candidate for the functional characterization of tau-related pathology.

### Strength and limitations

This study represents, to our knowledge, the first systematic description of cognitive vergence and pupillary temporal features as functional oculomotor signatures differentiating biological profiles defined by AT(N) criteria during an attentional task. The hierarchical analytical strategy, spanning continuous signal dynamics, trial-level feature decomposition, and subject-level analyses, provides a multi-resolution characterization that is more informative than any single level alone. The large number of temporal observations per participant supports the reliability of the extracted features, and the consistency of findings across analytical approaches strengthens confidence in the main observations. The use of AT(N) biological profile classification based on CSF markers, rather than clinical diagnosis, aligns with current frameworks and reduces the confounding influence of cognitive heterogeneity on oculomotor findings.

Important limitations must be acknowledged. The sample size is small and imbalanced (12 A−T+ vs. 26 A+T+), constraining statistical power and limiting generalizability. Although our analytical approach, including Firth-penalization and the leave-one-participant-out stability checks reported in the “Functional oculomotor signatures: trial-level features” section, was specifically selected to mitigate the risks associated with this design, independent replication in larger, balanced cohorts remains an essential next step before these findings can be considered generalizable. The cross-sectional design precludes any inference about whether oculomotor timing differences precede, accompany, or follow specific stages of pathological accumulation. The absence of a cognitively unimpaired control group prevents characterization of normative oculomotor timing in this paradigm, which would be needed to determine whether the observed patterns represent deviations from typical aging or reflect pathology-specific alterations. Medication use (e.g., cholinergic or adrenergic agents) could not be obtained due to ethical and data-sharing restrictions at participants’ referring clinical centers, and autonomic dysfunction, which can also influence pupillary responses, was not assessed; both remain possible sources of unmeasured variability. Longitudinal designs with larger and more homogeneously characterized samples, including cognitively unimpaired individuals at preclinical stages, are needed to determine whether these temporal oculomotor signatures track pathological progression and whether they are detectable prior to cognitive impairment.

## Conclusion

This study shows that cognitive vergence and pupillary temporal dynamics during an oddball task provide condition-dependent oculomotor signatures that differentiated A−T+ from A+T+ biological profiles in this MCI sample. The distinguishing information resides in the timing of peak responses rather than their magnitude, and the direction of profile differences reverses depending on whether stimuli require passive processing or active attentional engagement. These results may reflect the differential impact of isolated tau pathology versus combined amyloid-tau pathology on LC-mediated tonic modulation and cortical attentional network recruitment, with each profile showing its functional vulnerability under the conditions that most tax the neural systems it primarily compromises. Oculomotor signatures represent a promising and accessible complement to CSF-based profile characterization, and their confirmation in larger, independent cohorts, together with validation in longitudinal and preclinical samples, constitute meaningful next steps.

## Supplementary information

Below is the link to the electronic supplementary material.

ESM 1 (DOCX 51.5 KB)

## Data Availability

The dataset supporting the findings of this study contains sensitive clinical and neuropsychological information from a vulnerable population and is therefore not publicly available. Data may be made available upon reasonable request to the corresponding author, subject to ethical and institutional approval. Analysis code is openly available at https://github.com/ricardo-martinez-flores/ATN-oculomotor-profiles.
